# Primary Sjogren Syndrome: Focus on Innate Immune Cells and Inflammation

**DOI:** 10.3390/vaccines8020272

**Published:** 2020-06-03

**Authors:** Chiara Rizzo, Giulia Grasso, Giulia Maria Destro Castaniti, Francesco Ciccia, Giuliana Guggino

**Affiliations:** 1 Department of Health Promotion, Mother and Child Care, Internal Medicine and Medical Specialties, Rheumatology Section, University of Palermo, Piazza delle Cliniche 2, 90110 Palermo, Italy; chiararizzo87@gmail.com (C.R.); giuliagr09@gmail.com (G.G.); giuliadestro@hotmail.it (G.M.D.C.); 2 Department of Precision Medicine, University of Campania “Luigi Vanvitelli”, Via L. De Crecchio 7, 80138 Naples, Italy; francescociccia@tiscali.it

**Keywords:** Sjogren syndrome, innate immunity, inflammation, IFN signature, cytokines, innate lymphoid cells

## Abstract

Primary Sjogren Syndrome (pSS) is a complex, multifactorial rheumatic disease that mainly targets salivary and lacrimal glands, inducing epithelitis. The cause behind the autoimmunity outbreak in pSS is still elusive; however, it seems related to an aberrant reaction to exogenous triggers such as viruses, combined with individual genetic pre-disposition. For a long time, autoantibodies were considered as the hallmarks of this disease; however, more recently the complex interplay between innate and adaptive immunity as well as the consequent inflammatory process have emerged as the main mechanisms of pSS pathogenesis. The present review will focus on innate cells and on the principal mechanisms of inflammation connected. In the first part, an overview of innate cells involved in pSS pathogenesis is provided, stressing in particular the role of Innate Lymphoid Cells (ILCs). Subsequently we have highlighted the main inflammatory pathways, including intra- and extra-cellular players. A better knowledge of such processes could determine the detection of new therapeutic targets that are a major need for pSS.

## 1. Introduction

Primary Sjogren Syndrome (pSS) is an autoimmune exocrinopathy, characterized by xerophthalmia end xerostomia, and caused by a chronic inflammation of lacrimal and salivary glands. Moreover, pSS can display systemic features affecting extraglandular sites such as joints, vessels, lungs, nerves and kidneys [1]. This chronic inflammatory disease can lead approximately 5% of patients to a severe hematological complication such as B-cell non-Hodgkin’s lymphoma. This unfavorable event is mainly due to the hyperactivation and the concomitant disruption of adaptive immunity, as well as to the persistent inflammation at the tissue level [2]. pSS is more common in women, as with most autoimmune diseases, with a ratio of 9:1 females to males. The prevalence of this disease is about 0.5% with a typical onset of symptoms in middle-age individuals, usually between 40 and 60 years old [3].

The pathogenesis of pSS relies on a complex interplay between both innate and adaptive responses, causing the outbreak of autoimmunity characterized by the loss of self-tolerance.

Up to date, literature has focused attention on adaptive immunity in pSS, describing the network that determines the production of autoantibodies known as the hallmark of this rheumatic disease: anti-Ro and anti-La. However, in the last few years, a growing body of evidence has pointed out the importance of innate immunity in the earlier stages of pSS and in sustaining the pro-inflammatory milieu in the targeted tissues [4]. A better understanding of these mechanisms is required to plan future research in order to eventually identify new therapeutic strategies.

The present review aims to clarify the role of innate immunity in pSS development, taking into account all the evidence delivered by the most recent literature. The attention will be focused on cells, with a specific interest on new subsets such as innate lymphoid cells (ILCs) and the molecular mechanisms activated by their stimulation.

## 2. Innate Immune Cells in pSS

In a physiological condition, innate immunity is implicated in the first line of defense, especially at the epithelial level, and this is necessary for identifying several microbial components. These Pathogen Associated Molecular Patterns (PAMPs) are recognized by pattern recognition receptors (PRRs), expressed on innate cells [5]. However, exogenous antigens are not the only agents that can activate innate immunity; self-antigens also stimulate innate immunity by binding toll like receptors (TLRs), which belong to the super-family of PRRs. The consequence in pSS is the production of high level of Type I Interferon (IFN), the signature cytokine in this condition [6,7].

**Figure 1:**
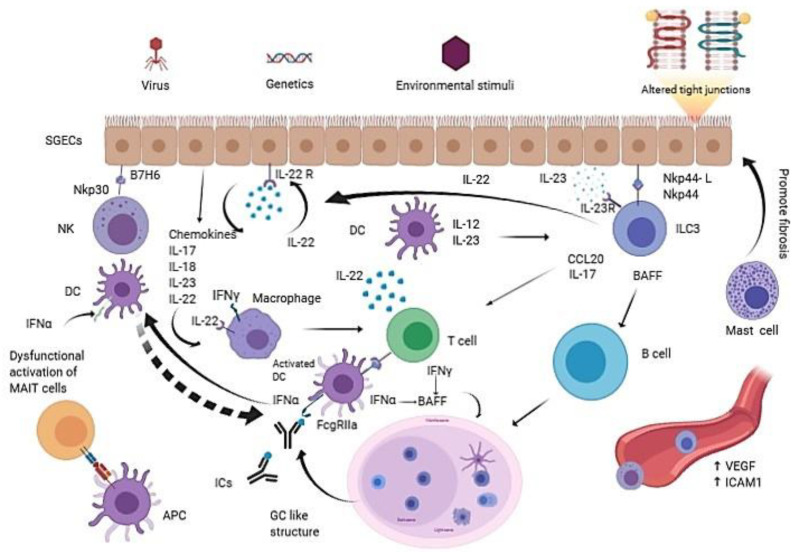
The interplay between innate immune cells and the inflammation prone microenvironment in Primary Sjogren Syndrome (pSS). pSS is a multifactorial rheumatic disease: environmental stimuli, in genetic susceptible subjects, may trigger Salivary gland epithelial cells (SGECs) to express ligands, receptors and cytokines, such as IL-22, that act in a paracrine and autocrine way when determining the activation of several innate immune cells like NKs, ILC3s, DCs and macrophages. SGECs exhibit a subverted architecture mainly characterized by altered tight junctions. The pro-inflammatory milieu, boosted by a huge production of cytokines and chemokines, promotes the recruitment of more innate immune cells and finally drives the formation of GC-like structures, which are responsible for the in situ autoantibodies release. The aberrant production of VEGF determines chaotic neoangiogenesis; activated endothelial cells express ICAM-1 that mediates immune innate cells tissue infiltration. Mast cells contribute to fibrosis and fatty infiltration of salivary glands. MAIT cells display a dysfunctional activation with a consequent impaired production of protective cytokines. The overall immune response appears to be Th17 polarized, suggesting a pivotal role for this cytokine in pSS. APC: Antigen presenting cell; BAFF: B-cell activating factor; DC: Dendritic cell; GC: Germinal center; ICs: Immunecomplexes; ICAM-1: Intercellular adhesion molecule 1; IL-22R: IL-22 receptor; ILC3: Type 3 innate lymphoid cell; MAIT: Mucosa-associated invariant T cell; SGECs: Salivary gland epithelial cells; VEGF: Vascular endothelial growth factor.

A complete description of the main immune cell groups involved in Innate Immunity in pSS is found in Figure 1.

### 2.1. Dendritic Cells (DCs)

DCs are antigen presenting cells and represent immunological sentinels that operate to detect dangerous insults to tissues rapidly. Their role in activating adaptive immunity leading to the production of antibodies is well known. This process requires the activation of both follicular (FDCs) and plasmacytoid dendritic cells (pDCs), which were actually found to be significantly increased in pSS salivary specimens [8]. As previously mentioned, pSS is an autoimmune disease characterized by the presence of autoantibodies that are produced in inflamed salivary tissue after the formation of ectopic germinal centers.

The precursor population of DCs in pSS displays an aberrant phenotype. In particular, their migration to salivary gland tissue seems to be related to the upregulation of chemokine receptor CCR5 and with an increased level of CCR5 ligands (CCL3 and 4) on saliva of pSS patients [9].

In addition, DCs altered genotype in pSS can cause their premature apoptosis in peripheral blood of patients [10]. These observations, taken together may explain DCs’ increased number in pSS salivary glands and their reduction among circulating cells.

The activation of DCs in autoimmune epithelitis is related to exogenous triggers, such as viral infections accounting for the viral hypothesis in pSS pathogenesis. DCs react to dangerous stimuli via TLR activation, finally leading to secretion of type I IFN as well as other pro-inflammatory cytokines. Self-antigens, derived from epithelial cells apoptosis in a dysreactive immunity background, activate pDCs endosomal TLR-7 and 9, inducing the pro-inflammatory cascade [11,12].

In a minor way, other TLRs (2, 4 and 8) activated in inflammatory condition, were described on pDCs of pSS patients [13]. The key effector cytokine of pDCs stimulation, type I IFN, determines in autocrine and paracrine ways pDCs activation with a continuous reinforcing loop highlighting the importance of the IFN signature in pSS. Type I IFN activates monocyte circulating cells, inducing them to produce BAFF (B cell activating factor) that contributes to the production of pSS-autoantibodies, by linking pDC functions to autoimmunity outbreak [14,15].

FDCs are specialized reticular fibroblasts originating from local fibroblasts or fibroblast precursors activated in inflamed tissue. FDCs network plays a pivotal role in organizing the microarchitecture of ectopic GCs-like structure. In this milieu, FDCs are able to present immune-complexes (ICs), formed by Ag-Ab and complement, to B cells determining B cells activation and promoting B cell survival and proliferation. Differently from other APCs, FDCs can retain ICs long-term on their surfaces, constituting a persistent stimulus for memory B cells re-stimulation. Moreover, FDCs do not show phagocytic activity and lack lysosomes and lysozyme [16,17].

To conclude, as previously stated, pSS has a higher prevalence in women and interestingly, pDCs expression of TLR may depend on gender. TLR-7 coding gene (Tlr7) is located on the terminal region of the X-chromosome. In murine models of Systemic Lupus Erythematosus (SLE) the translocation of this region on the Y-chromosome constitutes the Y-linked autoimmune accellerating region (Yaa), that has been associated with severe renal manifestation of disease in male mice. In particular, the sole duplication of Tlr7 is sufficient to accellerate autoimmunity. This observation suggests the possible implication of Tlr7 and Yaa in autoimmune disease pathogenesis and deserves to be further elucidated [18].

TLR-7 expression may depend even on sex hormones levels. Particularly, androgens, both in blood and tissues, affect pDCs, causing a decrease in the expression of TLR-7 and -9. Androgens protect epithelial cells from apoptosis, reducing autoantigens release and preventing the upregulation of TLR. In women, a low level of such hormones, as seen during menopause or as a consequence of defective local production of androgens, determines a constitutional higher expression of TLR in pDCs predisposing their hyperactivation and a possible consequent loss of immunological tolerance [11].

### 2.2. Macrophages

Macrophages are tissue resident innate cells involved in early defence against pathogens and in principal mechanisms of tissue homeostasis. These cells are characterized by significant plasticity, as their polarization and activation are determined by a combination of several environmental stimuli. At the beginning of the century, two extreme functional phenotypes of macrophages were described: M1/M2. M1 CD68^+^ promoted inflammatory response that was secondary to microbe attack, while M2 CD163^+^ contributed to the repairing of tissue damage, as well as promoting angiogenesis and removing cell debris through phagocytosis [19]. M1 CD68^+^ are drivers of a prevalent pro-inflammatory response, as they act as APC to T cells through the complex antigen-type II Major Histocompatibility Complex (MHC II) and directly produce inflammatory cytokines. On the other hand, the same cells are able to prevent dysreactive immunity by means of phagocyting impaired T-cells.

This dualistic function makes the understanding of the involvement of macrophages in inflammatory diseases such as pSS more complicated. It could be hypothesized that they rely on their ability to sense the environment and change consequently. As a matter of fact, these cells show a remarkable ability in shifting their functional phenotype from pro-inflammatory to protective, in relation to the microenvironment they are acting in. In the inflammatory setting, the expression of specific cytokines such as IFN, IL-12, IL-4 or IL-10 and the transcription factor HIF-1α (hypoxia-inducible factor 1 alpha) determine the polarization from one phenotype to another [19]. In conclusion, the biology of macrophages appears complex and dynamic due to the fact that these cells evolve in a heterogeneous continuum, resulting in high plasticity and reversibility so that any sort of strict categorization appears to no longer be suitable.

In pSS, salivary gland tissue macrophages appearance is an early occurrence; their number reflects the progression of the disease as it increases in later stages of this rheumatic condition [20].

Their function is related to the presence of IFNγ and IL-17 secreted by Th1 and Th17, respectively. These cytokines activate macrophages determining infiltration of salivary tissue, leading to exocrinopathy. Therefore, macrophages contribute to initiating and perpetuating the inflammatory epithelial damage.

Beside salivary glands, the epithelitis process may affect other epithelial barriers, as documented in eyes and kidneys. In particular, in ocular mucosa, immune cells infiltration occurs determining persistent inflammation. Chronic inflammation of eye epithelium determines the development of squamous metaplasia characterized by the keratinization of cornea surface, which represents the end stage of ocular involvement in pSS patients, who usually suffer from severe xerophthalmia. IL-1 secreted by activated macrophages has been demonstrated to play a crucial role in the development of squamous metaplasia. Experiments on eye tissue from murine models have demonstrated, not only the increase in number of macrophage in cornea and limbus, but also the aberrant network with CD4^+^ T cells. The latter, once activated, evolves in autoreactive clones that may perpetuate the activation of macrophages, themselves sustaining a pro-inflammatory auto-maintained loop [21,22].

In kidney specimens of patients who suffer from tubule-interstitial nephritis macrophages have been observed. Particularly, in a recent paper, the increase of HIF-1α as well as M2 CD163^+^ and M1 CD68^+^ was highlighted in renal tissue of pSS patients presenting hypokalaemia. In normal conditions M2 CD163^+^ cells and HIF-1α are not expressed in the tubule-interstitial space. This evidence may suggest a possible role for M2 in acute tubular injury and in the consequent restoration mechanism [23]. More studies should be conducted in order to clarify the protective action of these cells in renal damage during pSS and their possible use as therapeutic agents.

### 2.3. Mast Cells

Mast cells or mastocytes are cells rich in histamine and heparin granules that are usually found in the connective tissue. Their role in allergy and anaphylaxis has been extensively investigated. However, further research has underlined their possible involvement in tissue healing, angiogenesis and immune tolerance, as well as in defense against microbial insults.

In chronic inflammatory processes, mast cells contribute as key players and the first description of this subset in pSS dates back to 1998 [24]. Mast cells were demonstrated to promote fibrosis and fatty infiltration of salivary glands because of their interaction with fibroblasts and their contribution to collagen synthesis. Moreover, enzymes produced by mast cells can cleave and consequently activate matrix metalloproteinases (MMPs), which are considered as important mediators of tissue destruction and are produced by epithelial cells. Mast cells interact directly with the epithelium by releasing their activating enzymes after an early damage of epithelial barrier, as in the case of loss of basal lamina anchorage and the consequent altered cell adhesion. Therefore, the activity of these enzymes does not dependent on the lymphocyte infiltration but it precedes this phenomenon. Specifically, while analyzing salivary biopsies from pSS patients, increased levels of MMP-9 and -3 were detected; higher enzyme levels correlated with more structurally and functionally impaired glands were found as well, thus evidencing a more severe disease activity [25].

Recently, mast cells, identified using anti-tryptase antibodies, were observed in pSS salivary glands. However, a direct correlation between their number, angiogenesis and fibrosis was not established [26].

### 2.4. Salivary Gland Epithelial Cells (SGECs)

Salivary gland epithelial cells (SGECs) compose the ductal excretory structure and were considered classical non-immune cells in the past. Nowadays, thanks to several advances in understanding their functions, they are regarded as both the principal target of autoimmune responses and important players in the initiation of autoimmunity in pSS. In details, SGECs in pSS favor the inflammation process as a result of subverted cell architecture, cytokines production and immune cell recruitment; they simultaneously boost adaptive response, as they are the major source of pSS autoantigens, therefore acting as immunogenic elements [27].

In pSS, the onset of the inflammation process starts with the loss of polarity of SGECs [28]. Tight junction maintains cell polarity and in pSS several studies evidenced a dysfunctional expression of this molecules in SGECs. In particular, a decrease of occludin and Zo-1 and redistribution of claudin were described. This architectural disorganization has been linked to IFN and TNF levels thus suggesting a causative role for these cytokines. Clinically, the expression of the dysfunctional tight junctions could account for the reduced saliva production, one of the main symptoms in this disease [29,30]. At the beginning of the inflammatory process, they express and secrete different chemiotactic factors for lymphocytes such as CXCL12, CXCL13, CCL19 and CCL2 as well as adhesion molecules (ICAM-1), costimulatory molecules (CD80–CD86) and MHC II [31,32]. This complex interplay determines the recall, adhesion and activation of T and B cells at the inflammation site.

SGECs determine the maturation of naive CD4^+^ T-cells in T-follicular helper cells and are essential in the maintenance of germinal centers, as pre-clinical studies have demonstrated [33]. Moreover, they directly secrete cytokines (IFN, IL-1, IL-6, IL-7 and BAFF) involved in the constitution of a pro-inflammatory environment that finally leads to epithelitis [34,35].

Several hypotheses have been proposed to assess their role in pSS, but the most fascinating regards the involvement of the endoplasmic reticulum stress model [36]. In fact, SGECs have extended ER in order to produce fluids such as saliva and its proteins as typical secretory cells, found also in lachrymal glands, bronchioles, bile ducts and renal tubules. However, when the request of protein production is huge, the ER system undergoes a severe metabolic stress, evidenced by the dilatation of its lumen [37]. At this point, different mechanisms such as unfolded protein response (URP), autophagy and apoptosis emerge. In particular, the activation of URP, a process that needs high energy levels, determines the subsequent arrangement of autophagy-genes. Doing so the cell can obtain the metabolic support to maintain the URP process active [38,39]. When the stress trigger continues, it overcomes the possibility of the cells to survive causing apoptosis. Hence endoplasmic reticulum’s (ER) stress in SGECs can trigger the immune cascade. In particular, several products of the ER stress can activate immune response such as IL-6, produced during URP activation; MHC expressed during autophagy process; and nucleic acid exposed on cell surfaces into apoptotic blebs [40].

In addition, clarification about the link between ER stress and the expression of pSS autoantibodies (anti-Ro and anti-La) was provided [41]. During ER stress, in the course of apoptosis, cytoplasmic antigens (Ro and La), move into the cell membrane and in the final stage of this process are exposed outside the cell into apoptotic blebs. Therefore, the interruption of autophagy and the consequent aberrant cell death represent the trigger for an immune stimulation, finally determining the exposure of pSS self-antigens.

Hence, the apoptosis can no longer be considered as a “silent” process, since the discharge of self-antigens by apoptotic cells activates pathological autoimmune responses [42].

IFN acts as a major player among the cytokines produced by SGECs, even if the exact mechanism of IFN production is still unknown. The higher and dysfunctional expression in several autoimmune diseases, such as pSS, of Viral-like Long Interspersed Nuclear Element-1 (LINE-1, L1) that are highly repetitive DNA sequences, leading to the production of a huge amount of Type I IFN, has been suggested [43]. Human L1 contains two reading frames encoding for L1 ORF1p and L1 ORF2p proteins. The latter, physiologically expressed in germinal and somatic cells, has enzymatic activities that are essential for L1 functions [44]. The increased level of L1 ORF2p in ductal salivary cells may account for L1 hyperactivity with a consequential intensified production of IFN [45].

Another important finding is the lack of L1 ORF2p in high lymphocytic focus score biopsies of pSS patients and the increased expression of L1 ORF1p in high-grade B cell lymphomas, meaning that the reduction of ORF2p in pSS could be considered an alert for lymphomas development [46].

Instead, in low grade focus score a positive correlation with L1 ORF2p level and negative correlation with APOBEC3B, an inhibitory enzyme acting directly on L1, was found, suggesting a dysfunction in the inhibitory mechanism [45]. Conversely, SGECs apoptosis could expose L1 ORF1p and ORF2p to the immune system through the creation of ICs that, as mentioned above, directly activate pDCs and determine the production of type I IFN [43].

Taken together, these findings suggest a complicated mechanism beneath the immunogenic role of SGECs, which needs to be further investigated. At present, we can assume that chronic inflammation in a specific genetic background, could lead to an unbalanced antigens expression and cytokine profile in SGECs that may contribute to pSS development as well as to proliferative disease occurrence. In fact, SGECs may exert a pro-oncogenic role contributing to the development of lymphoepithelial sialadenitis (LESA), a pre-cancerous lesion associated with a high risk of developing MALT lymphomas. LESA constitution is due to the aberrant cross-talk with localized lymphocytic aggregates, but its exact mechanism is at the moment under investigation [47]. The complete knowledge of this process can lead to the development of new therapeutic intervention and may give physicians tools to interfere in this pathological feed-back in pSS.

### 2.5. Endothelial Cells

Endothelial cells, expressing CD31, constitute blood and lymphatic vessels. High endothelial venules represent the highways through which immune cells arrive at the inflammation sites, while the lymphatic vessels promote the arrival of antigens and APCs by the drainage of interstitial fluid into the tertiary lymphoid sites (TLSs), typically found in pSS salivary glands. These latter, differently from secondary lymphoid organs (SLOs) such as lymph nodes, spleen, tonsils and Peyer’s patches, lack in T and B cells organization, capsulation and vascularization [48].

The activated endothelium expresses adhesion molecules such as ICAM-1, VCAM-1 and E- and P- selectins necessary for the interaction with lymphocytes. In pSS patients the activation of endothelial cells is increased allowing an important migration of immune cells in inflamed tissues [49,50]. Moreover, the expression of ICAM-1 positively correlates with focus score of salivary biopsy [51]. Furthermore, in pSS, as well as in other chronic inflammatory diseases, an increased neoangiogenesis is observed. The leading cause relies on the overexpression of vascular endothelial growth factor (VEGF), that determines chaotic neoangiogenesis. Dysfunctional vessels lead to persistent extravasation of immune cells [52]. In addition, defective lymphatic vessels, characterized by over expression of CCL21, together with a dysregulation in chemokine gradient, increase the arrival and permanence of lymphatic cells into the TLSs [53].

We can conclude that even a dysfunctional endothelial activation may contribute to pSS pathogenesis and a deeper study of its mechanism is awaited to develop new therapeutic strategies.

### 2.6. Mucosa-Associated Invariant T (MAIT) Cells

Mucosa-associated invariant T (MAIT) cells are described as a specific subset of non-conventional/innate like T cells and represent a bridge between innate and adaptive responses [54].

As conventional T cells, MAIT cells originate from thymus, but differ by expressing an arranged TCR composed of Vα7.2 and Jα33 segments. Furthermore, they share with NK cells the expression of CD161 marker (CD161^high^/TCRVα7.2^+^ MAIT cells).

Formerly, this subset of cells was considered as T-CD8^+^, able to recognize vitamin B related peptides, expressed on pathogens through MHC class I molecules [55,56]. But subsequently the capacity of diversification in different T cell subsets was evidenced; this was underlined by the ability of MAIT cells in expressing both CD4 or CD8 co-receptors in order to respond to different stimuli [57]. In fact, the expression of CD8 enables MAIT cells to recognize intracellular peptides, meanwhile the CD4 receptor orchestrates a fast response to extracellular attacks [58,59].

Physiologically, MAIT cells are “natural memory” cells and promptly produce Th1, Th2 and Th17 cytokines under microbial insults [60]. Moreover, they represent potent regulatory cells able to maintain self-tolerance and homeostasis. Among MAIT cells, a specific subpopulation named CD161^dim^/TCRVα7.2^+^ T cells was initially described. They resembled MAIT cells without the CD161 receptor and were characterized by a reduced ability in cytokine production, compared to classical MAIT cells [61].

MAIT cells in pSS are involved in the dysregulation of the mucosal environment beneath the autoimmune epithelitis development.

In the first study describing the pathogenetic role of MAIT cells and CD161^dim/^TCRVα7.2^+^ T cells in pSS, the decreased number of both subpopulations in peripheral blood was evidenced, while their presence was observed in salivary gland tissue, due to the homing process [62]. Moreover, the altered function of remaining circulating MAIT cells has been revealed. MAIT cells and CD161^dim^/TCRVα7.2^+^ T cells detected in peripheral blood from pSS patients were mainly CD4^+^ and naïve, in contrast with controls. This might justify their reduced anti-microbial properties and their altered function determining the dysregulation of mucosal environment. In fact, both MAIT cells and CD161^dim^/TCRVα7.2^+^ T cells share a decrease in the expression of activation markers CD154 and CD69, as well as a reduction in TNF and INFγ production.

In conclusion, the functional role of CD161^dim^/TCRVα7.2^+^ T cells needs to be further elucidated in order to clarify their possible role in pSS aetiology, as they have been claimed to be an autonomous cell group independent from MAIT cells [63,64].

### 2.7. Natural Killer Cells (NKs) and Natural Killer T Cells (NKTs)

Natural Killer cells (NKs) represent the link between innate and adaptive immune system through their crosstalk with DCs and the interaction with epithelial cells. They are mainly involved in antiviral and anti-tumoral responses. In particular, classical NKs express the NKp30 receptor recognized by DCs with consequent production of IFN and IL-12. To date, contrasting evidence shows a possible pathogenetic role of these cellular groups in pSS. In mouse models, their activation appears protective towards pSS development and even helps in mitigating sicca symptoms. On the other hand, in pSS patients the presence of NK in salivary gland is correlated to local inflammation. The reason for this difference seems to depend on an increased NKp30 receptor expression. In support of this evidence, genetic mutation of the promoter of NKp30 gene, with a reduced transcription and transduction of the relative protein, determines the amount of protection from pSS [65].

The over-expression of NKp30 and its ligand (B7H6) on SGCE membranes at salivary level in pSS patients may explain the hyperactivity of NKs in the early phases of the disease, causing their aberrant cross-talk with SGECs and DCs [65]. The hyper-expression of B7H6 may determine the homing of these cells in pSS target tissue and could justify the decreased number of these cells in a pSS patient’s circulation. The NKs isolated from pSS blood patients, moreover, showed a dysfunctional phenotype with a lowered killing activity and a reduction in the expression of activating receptors [66].

NK functions may possibly be related to the inflammatory milieu in which they act in, and even depend on the stage of the disease.

An interesting review of our group described the pathogenetic role in pSS of a specific subset of NK cells named Natural Killer T cells (NKTs), focusing in particular on invariant NKT (iNKTs). iNKTs have been described as potential therapeutic agents or possible predictors to response to treatment in pSS. An attempt to use iNKTs as cellular therapy has already been reported in Systemic Sclerosis, and this evidence should encourage researchers to investigate iNKTs functions in pSS [67].

The NKTs, physiologically involved in immune responses against Gram-negative pathogens, share elements of both T cells and NKs and are able to discriminate self and non-self-antigens. Furthermore, they secrete INF and IL-4. The latter represents the hallmark of this subgroup of cells.

Three types of NKTs are known so far. Type I NKTs or invariant NKTs (iNKTs) express different activation receptors as semi-invariant T cell receptor (TCR) and CD161 (typical of NK cells) [68].

The attachment of TCR to the complex CD1d-glycolipids, expressed on B-cells, determines iNKTs activation and at a later stage, the production of both Th1 and Th2 cytokine profiles [69].

Alternatively, iNKTs, by linking with CD1d, suppress the B-cell auto-reactivity. Indeed, the lack of CD1d causes B-cells hyperactivation and a greater release of autoantibodies, suggesting that iNKTs play an immune-regulatory role in the maintenance of self-tolerance [70].

Several authors have tried to describe the activity and the pathogenetic role of NKTs in pSS.

A decreased number of NKTs in peripheral blood of pSS patients was found, possibly due to the homing in inflamed salivary glands or to apoptosis [71]. In addition, the reduction of circulating iNKTs was observed too, together with the increase of auto-reactive B cells at tissue level [72].

In spite of that, an increased number of iNKTs in peripheral blood of our pSS patients was detected, together with a lowered expression of chemokine receptors on iNKTs surface (CXCR3, CCR6 and CCR5). This evidence may justify a dysfunctional activity of iNKTs that may explain a diminished migration of these cells to the inflamed salivary glands and the absence of their protective activity against in situ autoantibodies production [73].

In conclusion, a former subset of NKs named NK22, characterized by the production of high amount of IL 22, has been recently recognized as a completely different cell group belonging to Innate Lymphoid Cells (ILCs), which are described in another section of this article [74]. The pathogenetic role of IL-22 in pSS patients has clearly emerged in the last few years; the overexpression of genes implicated in the production of this cytokine and its receptor (IL-22R) expressed on SGECs has been demonstrated and a positive correlation between salivary inflammation and level of IL-22 has been described [75].

### 2.8. Innate Lymphoid Cells (ILCs)

Innate lymphoid cells (ILCs) belong to a relatively new subsets of cells that seem to play a crucial role in the pathogenesis of several chronic inflammatory diseases [76,77]. Three different subsets of ILCs have been described so far. Each group is characterized by the expression of specific surface markers, cytokine production and depends on different transcription factors to develop and acquire functions. Specifically, T-bet, Gata3 and RoRγt are the master regulators of ILC1, ILC2 and ILC3, respectively [78]. Effector cytokines produced from each subset are IFNγ for ILC1; IL-4, IL-5, IL-9, IL-13 and GMCSF for ILC2; IL-17A/F, IL-22, IFNγ and GMCSF for ILC3 [4,5]. It shows clearly that ILCs share common feature, especially considering cytokine production, with their adaptive counterpart belonging to the T helper family, namely Th1, Th2, Th17 [79,80].

ILC cells can switch their transcriptional program on specific environmental stimuli gaining effector functions of a different ILC group. This phenomenon described as ILC plasticity deserves a deeper investigation to better understand ILC activity [81,82].

These cells are involved in protective processes against pathogens as viruses, bacteria and parasites as well as in granting tissue homeostasis [83]. However, their unbalanced activation in a pro-inflammatory milieu strongly participates in the dysreactive process beneath autoimmune diseases. In particular, there have been different reports accounting for a possible role of ILCs in inflammatory bowel diseases, rheumatic diseases, asthma and allergy [84,85].

In rheumatology, the analysis of synovial fluid from rheumatoid arthritis and psoriatic arthritis patients has revealed an increase in ILC1 and ILC3. ILC3 in particular are a major source of IL-17 and IL-22 [86]; these cytokines drive inflammation in spondyloarthritis and an increase of ILC3 was documented in psoriatic arthritis, as well as in ankylosing spondylitis [87].

ILCs are scarcely detected in blood, they can be depicted as “tissue resident cells”, mainly localized at epithelial barriers. Most of the evidence on their activity derives from studies conducted at the gut level that have extensively explored ILCs interaction with other immune cells and their response to microbiota stimulation [88,89].

In recent years, a growing interest in these cells has led researchers to explore their role, even in pSS. ILCs are developmentally and functionally related to lymphoid tissue inducer cells and their contribution seems essential to the formation of GCs-like structures as found in salivary glands affected by pSS [20,21]. Their constitution leads to local production of autoantibodies that finally contribute to the complete outbreak of autoimmunity typical of pSS [90].

In salivary glands, specimens ILC1 have been described, but a clear role for these cells has not emerged. No data are available for ILC2 in pSS.

However, in the last few years, IL-22 and related pathways have been highlighted as possible drivers of inflammation in pSS, therefore many efforts were made to identify the source of this cytokine and its related network [91]. In 2009 the first report of a new subset of IL-22 producing cells, defined initially as NK-22, in human tissues was provided. In particular, the authors described these cells in mucosa-associated lymphoid tissues (MALT) and demonstrated their activation when exposed to IL-23 [92]. NK-22 were further characterized at epithelial sites, especially in human tonsils, and thanks to a deeper investigation on their surface markers and gene expression panel, they could be more precisely recognized as ILC3 cells [93].

In particular, expression of IL-22 was evidenced to be strictly associated with the presence of NKp44 receptors on cell surfaces. NKp44 belongs to the family of NK receptor (NKR), and characterizes ILC3 NKp44^+^ cells [71] Its activation, due to environmental stimuli, triggers RORγt on these ILC3s that finally contribute in orchestrating a complex pro-inflammatory program [94,95].

In this regard, higher levels of IL-22 in pSS patients were detected when compared to controls. Such data showed a positive, statistically significant, correlation with clinical parameters of active pSS as saliva flow rate, autoantibodies presence, hypergammaglobulinemia and rheumatoid factor [96].

Subsequently, the feasibility of salivary glands biopsies has encouraged researchers to investigate how cells producing IL-22 and IL-22 contribute to pSS pathogenesis, directly focusing attention at the tissue level.

In 2012, an increased amount of IL-22 expression both at the mRNA and protein level was highlighted in salivary glands samples obtained from pSS patients. NKp44^+^ cells were significantly expanded in same patients and produced a higher amount of IL-22 when compared to controls. Moreover, the number of NKp44^+^ cells and the level of IL-22 were strongly correlated with the disease severity evidenced in higher degrees of focus scores that well reflect tissue inflammation. The concomitant expansion of Th17 cells was evidenced. Thus, these two cell populations appear as a major source of IL-22 in pSS tissues [75]. IL-22, a member of the IL-10 family, may play a protective versus a pro-inflammatory role depending on the microenvironment it acts in. In an inflammatory condition, the concomitant presence of IL-17 promotes a synergistic interaction with IL-22 thus causing chemokine production and immune cells recruitment that account for a predominant inflammatory scenario [97]. In pSS, this co-expression has widely been demonstrated, suggesting once again a probable pathogenetic role for this cytokine and consequently for ILC3 cells. Moreover, IL-22 is a downstream effector of IL-23; this cytokine was demonstrated to be highly produced in pSS salivary glands and interestingly IL-23 is involved in NKp44^+^ ILC3 development [92]. ILC3 are present at the epithelial interface in pSS and IL-22 is known to act through a specific receptor, IL-22R1, which is selectively expressed on epithelial cells [98]. A physiological antagonist, named IL-22BP, counterbalances IL-22 functions [99].

In pSS, a marked disequilibrium between IL-22 and IL-22BP was demonstrated. Moreover, an aberrant expression of IL-22R1 on hematopoietic cells was described in both peripheral blood and salivary gland tissue. In addition, the IL-22 axis, the aberrant and hyperexpression of IL-22R1 were shown to be related to the presence of high amount of IL-18, a cytokine produced via inflammasome activation. Taken together, these observations point out that, at a salivary gland epithelial site, in a pro-inflammatory milieu as in pSS, IL-22 producing cells may play a pivotal role in amplifying a dysreactive immune response that causes final tissue damage [100]. The IL-22 network induces STAT3, AKT and MAPK pathways activation; these mechanisms will be overviewed in detail in a special section in this review.

However, the production of IL-22 from ILC3 does not justify their implication in the formation of GCs-like structure. ILC3 appear as a powerful link between adaptive and innate immunity. A more extensive analysis on their chemokine production has revealed that ILC3 secrete CCL20. This molecule displays a strong chemotactic activity on immune cells, particularly lymphocytes and DCs, giving rise to the formation of mucosal lymphoid tissue in the area surrounding the epithelial barrier where inflammation occurs in earlier stages of the disease. Furthermore, ILC3 produce a B-cell-activating factor (BAFF) that stimulates B-cells to proliferate, survive and organize in GCs-like structures where in situ antibodies production takes place [101,102].

The role of ILC3 in determining this inflammatory-prone microenvironment appears of paramount importance, but further studies are required in order to clearly outline their activity [103].

Aspects that deserve to be clarified are mainly related to the migration of ILC3 in salivary gland tissues, their role in forming TLS and their interaction with epithelial cells [104]. Specifically, ILCs express the NKp30 receptor on their surface, the same receptor can be found on NK cells. In pSS an increased expression of NKp30 has been evidenced. It recognizes the B7H6 ligand expressed on epithelial cells; this underlines how complex the interaction at epithelial barrier sites could be [65].

Defining the exact aetiology of pSS remains a great challenge. Innate immunity, with a particular focus on ILC3, IL-22 production and the consequent activated pathways should be regarded with special attention in order to better clarify disease pathogenesis.

## 3. Main Mechanisms of Inflammation in pSS Related to Innate Immunity

Innate immune cells interplay determines a complex network of pro-inflammatory mediators’ production and triggers several intra and extra cellular patterns involved in inflammation. The dysreactive setting typical of autoimmune diseases usually relies on a genetic background that predisposes individuals to develop an impaired inflammatory response. pSS does not differ from the majority of rheumatic diseases; its multifactorial aetiology requires external stimuli and genetic alterations. In the last few years, both aspects and their consequent pathways have drawn attention of researchers, in order to better understand pSS and to find out new therapeutic targets.

### 3.1. IFN Signature, TLRs Activation, Intracellular Pathways Depending on JAK/STAT and MAP/ERK

IFN family includes a group of cytokines, produced by several cellular groups, that are involved in inflammatory responses against microbial agents, mainly viruses. Nowadays, three types of INF are known: type I, II and III, which include IFNα and β; which are IFNγ and IFNλ, respectively. Each IFN group activates specific receptors leading to the upregulation of IFN specific inducible-genes (ISGs). Most evidence on the role of IFN in pSS regards type I and type II IFN; but recently a role for type III IFN has been hypothesized too [105,106,107].

In particular, pSS is associated with genetic polymorphisms and mRNA expression profiles that are indicative of an excessive innate and type I IFN immune response. The upregulation of ISGs induced by type I IFN is known as type I interferon signature [105]. However, type II IFN role has been only recently evidenced and it represents the unique IFN signature only in 7% of pSS patients, being usually associated with a stronger type I INF signature [108]. The main source of type I IFN are DCs and CD14^+^ monocytes; pDCs can also produce high levels of type III IFN. Both type I and III IFN transduce intracellular signals via STAT1 and STAT2 heterodimers, that determine ISGs expression through the binding with IFN-stimulated response elements. Despite sharing a common signaling pathway, type I and type III exert different biological activities because they interact with specific receptors expressed on different cells. In particular, type I IFN receptors (IFNAR) are found on all nucleated cells; while type III IFN receptors (IFN lambda receptor: IFNLR and IL10 receptor) are expressed almost exclusively on epithelial cells.

Type II IFN is produced by T cells, NKs and NKTs. IFNγ dimers bind to the receptor (IFNGR) on macrophages and naïve CD4^+^ T cells controlling their activation and differentiation. The downstream signaling involves STAT1 homodimers that interact with IFNγ activated sites (GAS) in order to promote ISGs expression [109,110].

In pSS a significative upregulation of type I IFN has emerged as a key feature of this disease and it is believed to play major role in its unbalanced immune response. The upregulation of type I ISGs was demonstrated in PBMCs, pDCs, B cells and salivary glands in pSS [111] and 50–80% of patients show a positive type I IFN signature [108].

It has been hypothesized that the initiating factor in activating type I IFN responses, in a genetically determined background, is the abnormal innate immune response against endogenous or exogenous antigens, such as viruses or nucleic acids derived from damaged salivary gland epithelial cells. This activation depends on TLRs and cytosolic sensors of nucleic acids stimulation usually expressed on pDCs. Both TLRs and cytosolic sensors of nucleic acids were demonstrated to be upregulated in pSS. Amongst the latter, retinoic acid inducible gene 1 (RIG1), melanoma differentiation associated protein 5 (MDA5) and retinoic acid inducible receptors (RLRs) seem to be the most implicated in contributing to a type I IFN signature in pSS [112]. Once activated, these receptors trigger an intracellular pathway that induces type I IFN production. This cytokine, in turn, stimulates IFNAR expressed on the same pDCs, causing ISGs expression. One of the ISGs produced is BAFF, which determines B cell activation into plasma cells that are responsible for autoantibodies production. Simultaneously, IFNα can exert a direct cytotoxic activity on cells and can even contribute to Ro52 expression. The consequent formation of ICs between autoantibodies and autoantigens can again activate TLRs via FcgRIIa receptor on pDCs depicting a complex auto-maintaining loop of pro-inflammatory activation characterized by a huge amount of type I IFN release [113,114].

To deeper describe TLRs activation is a good opportunity to underline that the first line immune response depends on exogenous or endogenous danger signals, named PAMPs and damage or danger-associated molecular patterns endogenous ligands (DAMPs) that include heat shock and ECM proteins [115]. DAMPs determine a sterile inflammation, without microbial infection, with a subsequent tissue damage. PAMPs and DAMPs are recognized by PRRs expressed on immune cells. Different types of PRRs are described as a TLRs family or NOD like receptors (NLRs) [116].

TLRs are demonstrated to be overexpressed in pSS salivary tissue and peripheral blood from patients and mouse models. A brief overview of TLRs and their related pathways will be discussed to better focus their role in pSS [117,118,119].

Several TLRs are dysregulated in pSS, most evidence concerns TLR-2 and 4 that are surface receptors [114]; differently TLR-3, 7 and 9 are located intracellularly. All these TLRs, except for TLR-3, activate a specific transcription pathway mediated through MyD88 (myeloid differentiation primary response 88), a ubiquitously expressed adaptor molecule used by all immune cells, that has been highlighted as a crucial signaling mechanism in rheumatic diseases, including pSS [120]. This protein activates Nuclear Factor-kB (NF-kB) to transduce inflammatory signals. MyD88 pathway leads to increased levels of Tumour Necrosis Factor Receptor-Associated Factor 6 (TRAF 6) and to the activation of Interferon Regulatory Factor 7 (IRF 7). Their translocation in SGECs nuclei determines the production of IFN [121,122].

Other direct targets of TLR signaling are MAPK/ERK and JAK/STAT networks that additionally contribute to an IFN signature in pSS [106].

Cell surface TLR-2 and TLR-4 are expressed on peripheral blood mononuclear cells (PBMCs) and their stimulation leads to the promotion of Signal Transducer and Activator of Transcription (STAT3) and NF-kB pathways and consequently to the discharge of high levels of pro-inflammatory cytokines, as IL-17 and IL-23 via IL-6. Furthermore, the stimulation of SGECs with lipopolysaccharides and peptidoglycans (ligands of TLR-2 and TLR-4, respectively) determines the upregulation of co-stimulatory molecules such as ICAM-1 and MHC I [119,123].

A study published in 2012 highlighted the role of IL-23 and IL-6 IL-23 determines the Th17 polarization and the consequent IL-17 production via TLR-2 stimulation in PBMCs obtained from pSS blood samples. Moreover, IL-23 antibodies inhibited the stimulatory effect of TLR-2 blocking IL-17 production but did not affect the production of other cytokines like IL-23, IL-6, IL-1β and TNFα. Conversely, IL-6 antibodies blocked the production of IL-23, IL-1, TNFα and IL-17. In this case, an additional treatment with anti-IL23 did not demonstrate a synergic effect. These findings suggested that the production of IL-17, stimulated by TLR-2 activation, is mainly mediated by IL-6 in pSS [124].

The intracellular pathways triggered after TLRs activation and IL-6 production are related to NF-kB and STAT3. This was confirmed by the evidence of the high phosphorylation levels in salivary samples from pSS patients that justifies the hyperactivation of STAT3 and NF-kB. The latter activation is determined via the inactivation of its inhibitor (IBK) by phosphorylation [106].

Several studies in murine models and in humans demonstrate an important role of TLR-3, expressed in SGECs. Its stimulation, mediated by viral dsRNA, causes the production of type I INF and the upregulation of ICAM-1, CD-40 and MHC I. This is the only TLR MyD88-independent pathway. This receptor has been implied even in glandular apoptosis mechanisms but this observation needs further elucidation [120].

In addition, the activation of TLR-3 expressed by SGECs induces the production of BAFF, important in the activation of B cells, and in the production of an anti-Ro52 antibody [119,125]. TLR-7 and TLR-9 are expressed in PBMCs, B cells and salivary tissue from pSS patients. In B cells, their stimulation determines an increased induction in the phosphorylation of STAT3 S727, an epitope of STAT3, and NF-kB that was shown to correlate with autoantibodies release [106,126].

Recently, the phosphorylation level of the epitopes ERK and STAT were found to be upregulated in pSS patients versus healthy controls. In a pathological condition, a stronger intracellular signaling activation in NK cells, T and B cells was evidenced. Cytokine levels positively correlated to basal phosphorylation levels and the activation of these pathways was related also with different clinical manifestations. The activation of this axis contributes to a type I IFN signature [106].

Recently, in salivary gland specimens from patients with pSS, a predominant type II IFN signature was demonstrated. This observation is apparently in contrast with the well-known systemic type I IFN signature described so far. However, this difference could be related to distinct IFN activation patterns expressed at the local and systemic levels within the same patient. Studies aimed to analyze IFN signature both in salivary glands and peripheral blood from same subjects are needed in order to clarify this discrepancy and to better define the role of IFNs in pSS [107,127].

Besides IFN, the involvement of JAK/STAT pathway in pSS pathogenesis could be associated to the presence of reactive oxygen species (ROS). ROS contribute to phosphorylation of STAT3, which leads to the induction of adhesion and regulatory molecules such as ICAM-1 and PD-L1 in SGECs. ICAM-1 mediates lymphocytic infiltration while PD-L1 promotes SGECs survival, resistance to apoptosis and reduction of IFN produced by Th1 cells when bound to its ligand (PD-1) [128].

### 3.2. Inflammasome

Inflammasome is a multiprotein intracellular complex that plays a pivotal role in orchestrating inflammatory responses in a wide range of rheumatic diseases. The evidence of its activation at epithelial barriers has determined an increased attention towards its possible role in pSS [5]. Inflammasome is activated by the interaction of microbial components, toxins and mediators of cellular damage with PRRs. There are several types of inflammasome but in pSS most studies have focused attention on the NOD like receptor P3 (NLRP3) inflammasome. When activated, NLRP3 oligomerizes and recruits the adaptor protein, apoptosis-associated speck-like protein, and activates caspase. Expression of inflammasome genes such as P2X4, P2X7 (coding for purinergic receptors), NLPR3 and caspase-1 in pSS salivary glands positively correlates with focal lymphocytic sialadenitis and with autoantibodies production. This, once again, may suggest an intriguing scenario for the interaction between any innate and adaptive immune response in pSS. In particular, the P2X7 receptor (P2X7R), an ATP-gated ion channel connected with cell function as growth, apoptosis and neurotransmission, mediates the activation of NLPR3 and the P2X7R-NLRP3 inflammasome complex modulates the release of the inflammatory cytokines IL-1β and IL-18. These cytokines are responsible for the induction of pyroptosis, a specific immunostimulatory cell death, and have been demonstrated to be highly expressed in pSS [129].

These findings are supported by in vivo and ex vivo studies that evidenced higher expression of P2X7R in salivary glands specimens from pSS patient compared with healthy controls samples. In fact, mRNA levels for P2X7R were found to be elevated in pSS [130,131,132].

Moreover, a positive correlation between IgG, ANA and Ro/SSA and the increased expression of P2X7R mRNA was found in salivary gland tissue. The role of this specific inflammasome complex in pSS was further demonstrated in animal models. P2X7R deficient murine model does not develop salivary inflammation. Conversely, the addition of an agonist of P2X7R is sufficient to trigger inflammation in salivary glands from wild-type animal models [133].

The functioning of the effector cytokines produced by inflammasome activation is mediated by their specific receptors that have been demonstrated to undergo significant hyperactivation due to an exaggerate activity of their stimulating molecules. In order to explain this observation, according to recent literature, MyD88 is required for IL-1 receptor (IL-1R) and IL-18 receptor (IL-18R) functioning. IL-1 and IL-18 are elevated in exocrine tissue, saliva and PBMCs from pSS patients as well as MyD88 activity. Murine models knockout for MyD88 do not develop salivary gland autoimmune epithelitis, as demonstrated by preserved salivary flow [120].

### 3.3. Non Coding RNAs (ncRNAs) and Their Epigenetic Effect

Epigenetic mechanisms, such as DNA methylation and histone modifications could be implied in pSS pathogenesis. In addition, non-coding RNAs (ncRNAs) with epigenetic activity, may constitute a dynamic link between genome, environment and phenotypic manifestation of the disease. Several genes are involved in pSS development, such as HLA, STAT4, IRF and BLK (B lymphocyte kinase). In this rheumatic condition, genetic variants in the above mentioned loci are most often found in non-coding regions and may exert their effect on disease susceptibility by affecting the epigenetic machinery, which in turn modulates gene expression in target cells and tissues [134,135].

One of the most studied epigenetic mechanism is the transcription of ncRNA from intronic or intergenic genomic regions [136]. It acts as an important regulatory mechanism with implications in tissue differentiation, development, proliferation and cell metabolism. ncRNAs are classically distinguished in two main groups according to their length: microRNAs (miRNAs) and long-ncRNAs (lncRNAs) [137].

Aberrant expression of miRNAs has been linked to all complex autoimmune diseases, including pSS. The expression of miR-146a and miR-146b and their putative target genes IRAK1, IRAK4 and TRAF6 have been studied in PBMCs from patients with pSS and controls. Researchers found an overexpression of mirR-146a/b and TRAF6 in pSS, whereas IRAK1 expression was downregulated. However, data on miR-146 functions are contrasting. In fact, a role for miR-146 in reducing TLRs activity was documented; the decrease in its level should facilitate inflammation and autoimmunity outbreak. Nevertheless, as stated academically, the expression of miR-146 was elevated in both PBMCs and glandular tissue of pSS patients; thus suggesting a different activity of this miRNA in pSS that needs to be deeply investigated [138].

Others miRNA, as miRNA-155, have been studied with conflicting results in its expression level in pSS [139]. In addition, in 2015, the expression of a cluster of miRNAs was related to an increased presence of SSA/Ro and SSB/La in SGECs and PBMCs from pSS patients compared to non-pSS controls. This cluster, characterized by an upregulated expression, included miR-16 and miR-200b-3p in SGECs and miR-223 and miR-483-5p in PBMC [140]. The aberrant expression of self-antigens, due to miRNAs activity, triggers, in a pro-inflammatory dysreactive milieu, the production of autoantibodies.

Besides miRNAs, the role for most lncRNAs remains largely elusive. Emerging evidence has revealed that lncRNAs are involved in regulation of central cellular processes, such as genomic imprinting, RNA splicing, chromatin remodeling and protein transport, thus, suggesting a contribution of lncRNAs in the pathogenesis of autoimmune diseases [141].

A recent study identified more than a thousand lncRNAs in pSS salivary tissue. Their different expression showed a correlation with pSS manifestations including the presence of rheumatoid factor, anti-SSB autoantibodies and high titer of IgM. However, more research is needed to clarify their possible role in pSS pathogenesis [141].

## 4. Conclusions

A comprehensive knowledge of innate immunity activity in pSS may open new scenarios on possible future therapeutic approaches. Only preliminary studies have hypothesized treatment strategies targeting intra- and extra-cellular mechanisms in pSS and currently no drugs have been approved.

Considering the IFN signature, the most characteristic feature in this disease, it seems important to underline its possible role in clinical setting as a biomarker as well as a therapeutic target.

IFN upregulation correlates with severity in clinimetric indexes, such as Eular Sjogren Syndrome-Disease Activity Index (ESS-DAI) [108], suggesting that its evaluation could became helpful in routine clinical care. In particular, several reports have documented the possibility to stratify patients according to the prevalent IFN signature expressed. In fact, the different production of type I IFN, type I plus type II or type II only identifies subgroups of patients that present differences in clinical parameters, such as fatigue, and that can benefit from a selective IFN pathway blockade. Interfering with the IFN related-inflammatory cascade could be performed by directly blocking IFN or by inhibiting its production.

The first strategy has been attempted in SLE with monoclonal antibodies (mAb), as Sifalimumab and Rontalizumab that block IFNα, without significant results probably due to the absence of inhibition of other IFNs [142,143].

Meanwhile Anifrolumab, a mAb that blocks all type I IFNs, has shown more encouraging results in SLE. pSS lacks such studies, but they could represent a model for future research in this disease [144].

The second strategy is directed towards the cells responsible for type I IFN production and in particular pDCs. An experimental approach was attempted using RNases, that destroy the ICs known to activate pDCs, but no further research has kept investigating this approach in pSS [145,146].

Benefits regarding the eventual block of IFN production may derive from the inhibition of TLRs, Myd88 and kinases associated to IL-1 receptor. These targets are actually taken into account only for basic science research purposes, and are lacking in clinical setting trials [147,148].

A promising field in rheumatology is actually represented by small molecules. These new therapeutics show important advantages over biological therapies such as oral administration, pleiotropic effect and easier storage. Nowadays great expectations even in pSS are catalyzed in JAK/STAT inhibition. Some of the drugs belonging to this group are Baricitinib, Tofacitinib, Filgotinib among others. Some of them have been approved in inflammatory arthritis but several trials are ongoing in other rheumatic diseases, representing a valuable chance for pSS [149,150].

Thinking on cytokine blockade options, a feasible opportunity could be represented by IL-17 inhibiting agents. This possibility relies on the central role of IL-17 expression in cooperating to the production and to the activation of IL-22 and its related pathway, which has been demonstrated to strongly endorse inflammation in pSS. Trials attempting to assess efficacy of Secukinumab and Ixekizumab, mAb targeting Il-17, in pSS are awaited [151,152].

In conclusion, an ancient drug, hydroxychloroquine (HCQ), classically used in pSS to control arthralgia and cutaneous manifestations, has revealed its activity in the inhibition of TLR-7 and 9 activation. This activity is mediated by the direct binding of HCQ with TLR ligands, specifically nucleic acids. HCQ demonstrated to reduce gamma globulins levels and erythrocytes sedimentation rate in pSS but has failed in showing efficacy on main complains such as dryness, pain and fatigue [153,154,155].

## 5. Future Perspectives

Clearly more studies are needed to shed light on the all above mentioned mechanisms and possible therapeutic strategies. Innate immunity stands out as an innovative topic in pSS and its complete comprehension could be the source of novel targets, possibly leading to the development of efficacious treatments. Empowering the rheumatologist toolbox in pSS with target therapies tailored on patients represents a major unmet need in pSS and deserves future investigation.
